# Program Impact Pathways and Contexts: A Commentary on Theoretical Issues and Research Applications to Support the EsIAN Component of Mexico's Conditional Cash Transfer Program

**DOI:** 10.1093/jn/nxz221

**Published:** 2019-12-03

**Authors:** Jean-Pierre Habicht, Gretel H Pelto

**Affiliations:** Division Nutritional Sciences, Cornell University, Ithaca, NY, USA

**Keywords:** context, pathways, implementation research, Mexico, program theory

## Abstract

This commentary on the Integrated Strategy for Attention to Nutrition (EsIAN) journal supplement begins with a discussion about the challenges that implementation researchers confront with respect to analyzing complex impact pathways. We note that the research on the implementation of the EsIAN component of Mexico's conditional cash transfer program was based implicitly or explicitly on a program impact pathway approach, which used both quantitative and qualitative methods to examine bottlenecks in program implementation. We then identify 5 categories of contexts that affect the impact, implementation, and survival of intervention programs: *1*) biological, *2*) social-cultural, *3*) delivery modalities and platforms, *4*) bureaucratic, and *5*) political. Each of these contexts presents theoretical and methodological challenges for investigators. In this commentary, we focus primarily on biological and social-cultural contexts, discussing the theoretical and methodological challenges the investigators faced and the research strategies they used to address them, which have produced a unique compilation of “learning by doing” studies. We also touch briefly on the political context in which the Prospera program research was conducted. We conclude with statements that highlight the exceptional value of the journal supplement, not only with respect to the analysis of the interventions the studies cover and the sustained examination of a long-term program but also as a major contribution to the literature in implementation science in nutrition.

## Introduction

The journal supplement (1) presents the history of the research that informed, supported, and enabled the implementation of the health component [Integrated Strategy for Attention to Nutrition (EsIAN)] of Progressa-Oportunidades-Prospera (referred to here by its most recent name, Prospera, for simplicity)—a uniquely successful, large-scale, and long-term conditional cash transfer program. The supplement is a treasury of information and insights for theory and practice, particularly as the nutrition sector aims to achieve impact at scale (2).

Knowledge about effective interventions to improve the nutrition of mothers and their children has grown rapidly in recent decades, but how to scale them up to reach the populations who need them most has been slow. There is an urgent need to generate and effectively communicate the evidence that will support countries to achieve coverage and effectiveness of a range of nutrition-relevant actions and result in sustained impact on nutrition outcomes. Nutrition implementation efforts and researchers face daunting challenges in addressing these needs: *1*) nutrition interventions at scale are often embedded in broader programs and their pathways to impact are complex, *2*) they happen over long timeframes and in changing biological contexts, *3*) they cannot be easily stopped and started, and *4*) they often span large, diverse geographic and social-cultural areas. In this commentary, we discuss theoretical approaches to addressing some of the key challenges of doing nutrition implementation research at scale and examine how the investigators whose work is featured in the supplement addressed them in the context of Prospera.

### Challenge 1: analyzing complex impact pathways

#### Program impact pathway and program implementation pathway analysis

The systematic thinking that guided the research reported in the supplement is based implicitly or explicitly on a program impact pathway (PIP) (3). This concept is similar to the program theory that has been used to examine this and other cash transfer programs (4). A PIP approach for nutrition specifies the pathway by which interventions for an intended beneficiary (e.g., a child) progress through the “delivery system” of the program to the household and then within the household to deliver the interventions to the intended beneficiaries (5, 6). Some interventions, such as pills or tablets, remain unchanged as they progress through a PIP. However, others require additional actions (e.g., food preparations, modifications to feeding practices), and virtually all require knowledge transfer as well as other activities to motivate and support the behavior changes that are required. These are commonly referred to under the umbrella of “behavior change communication” or “social and behavioral change communication.” In addition to nutrition-specific interventions, a PIP often includes other supportive interventions, such as food preparation and storage materials, as well as information about them. These additional components enhance the use of resources and knowledge by program staff and by households. They may also be synergistic with the nutrition interventions.

In nutrition research, quantifying the flow in a PIP flow can be accomplished by structural path analyses to ascertain the magnitudes of mediation of the intervention from one pathway step to another. This can also be used to quantify “moderation”—which can be conceptualized as the role of facilitating and inhibiting factors on mediations. This is exemplified in the study reported by Zongrone et al. (7). The flow depends on the quality (Q) of the intervention. For many uses, these analyses require measurement corrections to deal with unreliability in the independent and mediating variables (8). Publications that use path analyses in nutrition program PIP-guided research have not consistently included the necessary reliability data (9, 10). This makes it more difficult to derive broader generalization.

PIP analysis can also be used to ascertain the coverage (C) of an intervention. Individual coverage is usually measured binomially (i.e., whether an intended beneficiary received the intervention with a specified level of quality). Population coverage is the proportion of beneficiaries who received the intervention at the specified quality level. Bottlenecks in program implementation are revealed by decreases in coverage at each sequential step as one proceeds along the PIP. This method can be extended to also identify the determinants of these bottlenecks (11).

In summary, we identify 2 components that quantify progress along the PIP steps. One quantifies the coverage (C) and the other quantifies the magnitude of the flow (Q) within the population covered. For example, Figure 1 in García-Guerra et al. (3) shows a PIP arrow from “Education sessions” to “Fortified Food Supplement.” Hypothetically, if 70% of the women participated in the education sessions (C) and there was an 80% improvement in fortified food supplement preparation (Q) among the women who attended the sessions, then the impact of education sessions (C × Q) would be 54%.

#### Contexts in PIP research

By context, we refer to the set of circumstances or characteristics in which an event, situation, or a program takes place. Differences in contexts or changes in a context foster or impede the flow of deliverables in a PIP. Context usually plays an important role at each of the junctions in a PIP. These can be identified in a model that depicts the transmission of critical elements of interventions.

**Table 1** identifies 5 categories of contexts we think are useful for classifying the factors that affect the impact, implementation, and survival of intervention programs. The biological context determines whether a child has a potential to benefit from the biological interventions. The social-cultural context affects the abilities of both the program delivery system and household delivery system to establish and maintain the behavioral aspects of the intervention. These 2 contexts permit, hinder, or foster the progress of interventions through the PIPs. The biological context of concern for a nutrition PIP is where the interventions are transformed intracorporeally into functional outcomes of health and growth. The social-cultural context is concerned with extracorporeal issues.

**TABLE 1:** Categorization of contexts that affect intervention programs

Context	Examples
1) Biological	Nutrition and disease conditions
2) Social-cultural	Social and ideational systems
3) Delivery modalities and platforms	Institutions, organizations, communication modalities
4) Bureaucratic	Organizational behaviors
5) Political	Policies reflecting values

In this commentary, we focus particularly on the biological and social-cultural contexts, as these received primary attention in the articles in the supplement. We also briefly discuss the fifth context—political context. However, we note that contexts 3 and 4 (Table 1) have also been addressed by the National Institute of Public Health of Mexico (INSP) research team. The delivery platform context (12) is represented by Bonvecchio et al. (13), and the fourth context (bureaucratic) is included in Gonzalez et al. (14).

Finally, it will also be apparent to readers of the supplement articles that the collaborations among ministries, research groups, and the Prospera program were not only continuously identified and examined over the years of activity, but these collaborations and the program itself depended on a supportive political context.

The following sections, which are devoted to challenge 2 and challenge 3, are structured in 2 parts. The first part examines the theoretical issues, and the second part discusses the context from the perspective of the Prospera studies in the supplement.

### Challenge 2: responding to changes in the biological context and potential to benefit from health and nutrition interventions

#### Theoretical issues

##### Efficacy and effectiveness

To facilitate the discussion of the biological context, we first need to review the concepts of efficacy and effectiveness within the context of a PIP model, particularly in relation to the fortified complementary food (Nutrisano) provided by Prospera. Biological efficacy is the final step in the PIP, which occurs after the intended beneficiary has consumed the nutrient or nutrient-containing food. It is the point at which the biological deliverables (e.g., nutrients) are transferred to the intracorporal flows that transmit them to the cells where they are metabolically active, as evidenced by measures of cognitive and physical health. Nutritional efficacy is a measure of the success of the intracorporal flow in achieving the nutritional changes that determine the desired health and cognitive outcomes.

A fundamental feature of biological efficacy in a population evaluation is that it depends on the intended beneficiaries’ capacity to benefit from the intervention—in the case of Nutrisano, the children's “potential to benefit” from the nutrition supplement.

Potential to benefit from a biological nutrition intervention has 3 prerequisites:

1. There is a need for more of the nutrient.
2. The nutrient in the supplement is appropriate to meet that need; in other words, it is efficacious.
3. No biological factors impede the nutrient's absorption and use so that the interventions can produce the desired health and nutrition outcomes (15).

To assess the efficacy of an intervention requires knowledge about the status of all 3 of the prerequisites in the population and using outcome measurements that reflect improved nutrition. Efficacy is investigated with a probability design (16) such as a randomized controlled trial (RCT) (17). This is used to show that the supplement groups benefit importantly and significantly (*P* < 0.05) compared with unsupplemented controls. Lack of potential to benefit because 1 or more of the 3 prerequisites are not present, or because of poor outcome measures, explains many disappointing evaluation reports.

With respect to Prospera, it is important to note that before taking the program to scale, its efficacy was ascertained with a randomized program evaluation study. The investigators examined the impact of Prospera in a randomized selection of villages that were similar to those in which the program would be scaled up nationally. This similarity is essential for foretelling the impact of the benefit nationally; it permitted a statistical probability statement about impact.

##### Ethical issues

For Prospera, as for most research that is undertaken to support programs, investigators are faced with several kinds of challenging problems, including challenging ethical problems. In an RCT, potentially valuable benefits are provided to the intervention group (e.g., children), which neither they nor the control children would have received if there was no trial. An RCT extending the benefits to everyone would eliminate the control group and would not serve the scientific purpose for which an RCT is undertaken. Prior to establishing the benefits of the intervention, the issue is one of “fairness” (18). Who is offered the potential benefits? In an RCT, the offerings are random, which seems fairer than any other allocation.

However, random allocation does not address other issues of fairness relative to the control groups who should receive benefits commensurate with their efforts. Another related principle that enters the discussion is the principle of beneficence, which is based on the proposition of extending benefits to everyone if it is feasible. Some RCT evaluations provide other types of benefits, such as medical care (19) to help ensure that the benefits to treatment and control groups are comparable. Thinking through beneficence for control groups (18) can result in better RCT designs for decision making because it may more effectively identify the relevant intervention and its consequences from other factors that are carried with the intervention (16). The ethical difference between “doing harm” and not providing beneficence is ill understood. This lack of understanding has led to the destructive concept of “equipoise” (20), where the control group must receive “state-of-the-art” treatment. The consequence of applying the concept of equipoise is that it results in not conducting ethical research to develop effective public health interventions. This is particularly the case for poor populations who need it the most. In these populations, the impact of importance is not relative to a state-of-the-art condition but relative to the usual condition, similar to that which one hopes a large-scale intervention will improve.

In contrast to providing *probability* evidence of efficacy for initiating a supplementation program, the challenge in an ongoing program is how to organize evidence to change or stop a supplement when changes in the biological context are likely. This requires a sequence that begins with evaluating the *adequacy* of program effectiveness and then examining the *plausibility* that changes in a supplement will change program adequacy. An adequacy evaluation ascertains whether the results are adequate relative to a standard without, however, ascertaining that they are caused by the program. Adequate results could, for instance, be due to secular changes in well-being that are not related to the program. Plausibility evaluation ascertains whether the results can plausibly be due to the program (16). Other, related issues (e.g., cost and acceptability) also are important for making decisions about to how to proceed programmatically.

The basis for a decision to change or stop a supplementation program is different from that used to initiate one. From a technical perspective, inferring similarity of effect in an RCT on the basis of not demonstrating a statistical difference is scientifically incorrect. It is highly susceptible to arriving at an erroneous conclusion due to inadequate sample sizes (e.g., 20, 21). Instead, one must show that the candidate supplements are not inferior to a currently acceptable one. This is currently ascertained through randomized controlled noninferiority trials (22). However, these trials are not ethical for most ongoing programs. Consider the problem of attempting a noninferiority trial with a control group, which requires comparing impact, for example, between supplemented and unsupplemented children in a situation where all children are already receiving the supplement. The unsupplemented control children would lose the supplement. This causes harm. It is “malfeasance” and unethical according to all the canons of research ethics.

Returning to our discussion of efficacy and effectiveness with respect to implementation research, it is essential to point out that efficacy is concerned with the impact of an intervention under ideal delivery circumstances, whereas effectiveness describes the impact under “usual” circumstances. In program-relevant PIP analysis of a supplement, effectiveness assessment focuses on programmatically relevant outcomes that measure adequacy because adequacy measures include both the “efficacy effect” and the “impact effect” of the supplement delivery through the whole program impact pathway. Effectiveness is concerned with achieving an intended physiological outcome in the population. It depends on both the efficacy (E) of the intervention, the quality (Q) of its implementation, and the coverage (C) of delivery of the intervention to those who can benefit. Efficacy includes the recipient's potential to benefit from the intervention. From the perspective of a biological outcome, effectiveness is calculated by multiplying E × Q × C. Effectiveness fails if either the quality or the coverage fails. This is often not well understood and, as a result, leads to lack of efficacy being blamed for poor effectiveness. This error can occur with any nutrition intervention but is especially the case when the intervention involves a supplement and the quality of delivery is neglected and not measured (23) or is measured inadequately (24).

In summary, the foregoing background discussion suggests the utility of the following algorithm to examine a fundamental issue of concern in many nutrition implementation research studies on supplementation—namely, whether to improve or to stop supplementation:

- Was the program adequately biologically effective as judged by international norms of healthy nutrition? This judgment about adequacy is independent of the probability (<0.05) of causality.
- Was this adequacy plausibly accomplished by the delivery (Q × C) and efficacy (E) of the supplement? For example, is there evidence that nutritional outcomes are a result of high adherence to recommended intake and known efficacy of the intervention and not due to changes in codeterminants of the nutritional outcome?
- Are there other considerations, such as acceptability to child and mother (7) and cost (i.e., context), that must be taken into consideration?

#### Biological context in the Prospera studies

Nutrisano, a whole milk–based fortified food, was developed by Mexican researchers based on local knowledge of nutritional status at the time and an extensive global evidence base. It was selected to be the nutritional supplement for Prospera. However, an initial evaluation in 1997 (25) indicated a lower biological impact than expected. That result suggested that either the “program deliverables” (knowledge and a nutritional supplement) had less efficacy than the previous research led the investigators to expect, or the program components were not as well delivered and used as had been anticipated. In the ensuing years, an ongoing research program to support the program by systemically parsing out where to look for inadequacies, identify problems, and develop solutions (13, 14) has been a hallmark of Prospera.

Turning to the articles in the journal supplement that are concerned with the biological context, it is important to note at the outset that INSP verified all the prerequisites for the efficacy of Nutrisano's iron and macronutrients before implementing the Nutrisano program widely. Potential to benefit from a national Prospera program was ascertained at the beginning of the program. Thus, they chose the nutritional supplements based on their demonstrated efficacy in improving the nutritional health of malnourished children.

However, over time, changes in dietary and anthropometric data in Mexico suggested that the potential to benefit from an energy-containing supplement might have changed since the beginning of the program 2 decades earlier. In fact, it looked as if the population was ingesting too much energy. In the case of Nutrisano, the lack of prerequisite 1, a need for more energy, would mean that Nutrisano would no longer be an efficacious intervention. Thus, substitutes that had the same micronutrient content, including iron and zinc, were considered. One of these substitutes, micronutrient powders delivered in sachets, was less than a quarter of the price of Nutrisano. Another substitute, syrup, cost more than Nutrisano.

The question for the researchers was whether 2 other supplements were not only as efficacious as Nutrisano but also as effective. The conventional approach to answering this question would be to compare Nutrisano's impact with that of the 2 other supplementation candidates in an RCT, using a probability design. However, as discussed in Neufeld et al. (26), this would have required depriving some children access to the supplementation, children who would otherwise have received it from the program. It was clear that there could be no control villages because the Prospera program was nationally implemented.

The researchers took an innovative approach, deciding to examine each of the 3 supplements for their individual abilities to meet adequacy of performance. To do this, the researchers began by randomizing a sample of the study population into 3 comparable groups, so that any conclusion about adequacy would be similar for all of them. The findings revealed that all of the groups were adequate. These analyses do not reveal why they were adequate but showed that the adequacy conclusion could be extended to the population the samples represented.

Several issues follow from the study:

1. With respect to the hemoglobin values, how can one support the conclusion that they were adequate in all 3 groups? In Neufeld et al. (26), we see that, according to the WHO norms (27) [Table 1 in Neufeld et al. (26)], the mean values were adequate, which means that all 3 supplements were adequately effective. Note that the comparison to the norms is with mean values and not with percentages of deficiency. This is parallel to comparing means of anthropometric measures rather than percentages of stunting and wasting (28).
2. A dose-response plausibility analysis (13) supported the inference that the adequacy in attained hemoglobin was due to the supplementation. This plausibility analysis (cf. 15) is made possible because some children did not ingest the full prescribed dose of the supplement. The dose-response analysis demonstrated that differences in reported intake of the supplement were significantly associated with hemoglobin values. This finding could be due to the possibility that more adherent mothers might also be providing better care and healthier diets that result in higher hemoglobin levels. However, the more plausible explanation is that the supplement was the cause of the dose response, thus fulfilling the “need for more of the nutrient” prerequisite for potential to benefit. Based on the evidence from the dose-response results, one can conclude that decreasing iron supplementation would decrease the adequacy of effectiveness. It also leads to the conclusion that one cannot ethically withhold the supplement.
3. The adequacy analysis showed some modest differences in hemoglobin values in the 3 groups. However, the dose-response analyses revealed that these differences in effectiveness were entirely due to the dose consumed. Thus, the equivalences of the 3 supplement formulations relative to iron nutrition were persuasively demonstrated without an RCT design.
4. Shifting from hemoglobin to growth, the other major nutritional outcome Nutrisano was designed to address, Neufeld et al. (26) found that levels of attained length at 24 mo were inadequate relative to the WHO norms (29). There was a mean deficit of 2.8 cm, with a mean attained length-of-age *z* score of −1.04 (*P* < 0.05). This is unacceptably low from a public health perspective. However, it is essential to note that the length deficiency was mostly established before the mean baseline age of 8.2 mo. After this age, the subsequent growth to 24 mo was almost adequate. This is indicated by a deficit in growth from baseline of only −0.06 *z* score, very close to a healthy zero *z* score. Also, the dose-response analysis indicated that those who did not consume the supplement grew only 0.7 cm less well from baseline than did those who ingested the prescribed dose, a negligible difference from zero. Finally, the weight-for-length and BMI data did not reveal evidence of inadequate energy intake.

Taken together with the findings enumerated above, along with results from other Mexican epidemiological data, the investigators’ conclusions confirm that the macronutrients in Nutrisano were no longer necessary for children older than 8 mo (the mean age of entering the adequacy evaluation study). On the other hand, the study also clearly highlights the inadequacy of infant attained growth. This latter finding raises other important questions. For example, is it due to the intergenerational effect of maternal childhood stunting or to inadequate infant feeding (30)?

In summary, the algorithm we suggested in the background section above was fully and effectively used in the outstanding research reported in the journal supplement. It supports the following conclusions:

1. Adequacy evaluation showed that micronutrients were deficient between baseline (mean age 8.2 mo) and 24 mo of age.
2. Adequacy evaluation showed that macronutrients as measured by growth were adequate between baseline (mean age 8.2 mo) and 24 mo of age.
3. Attained growth is not adequate in the early months of life (before baseline).
4. Plausibility evaluation by dose response showed that for both anemia and growth outcomes, effectiveness depended on consumption, not on differences in efficacy among the 3 supplements.
5. These findings permitted the recommendation to halt the use of Nutrisano and choose a much less expensive supplement with lower distribution costs, which could also be fed more frequently, thus increasing the likelihood of consuming sufficient quantities to meet children's nutrient requirements. In other words, more ingestion with equivalent efficacy would lead to greater effectiveness.

The Prospera studies demonstrate the utility of applying a decision-making algorithm to inform decisions about whether to eliminate a widespread supplementation program or whether it is essential but needs to be modified to meet changing population needs and conditions.

### Challenge 3: accounting for heterogeneity in the social-cultural context of the program and household implementation

#### Theoretical issues

By “social-cultural context,” we refer to a wide range of factors that are involved in the organization and function of human activities, specifically from the perspective of implementation research in nutrition, involved in access, acquisition, and consumption of foods and nutrients. These factors moderate the effectiveness of an intervention (31) through enhancing or obstructing the flow and coverage of the PIP step. In a nutrition intervention, they do not usually add directly to the interventions (e.g., by providing more supplements or informational content). As was the case in Prospera, in addition to providing motivation, the informational component of an intervention is often designed to expand maternal knowledge to the knowledge that has been acquired through other routes (e.g., schooling) to enable caregivers to make better use of all household resources, including the supplement.

In a PIP analysis, whether it is undertaken for purposes of intervention planning, formative research, or process evaluations, the social-cultural context is pertinent as the focus of research activities to identify and address the economic and social structure of both the nutrition intervention delivery system and the household delivery system. With respect to the household delivery system, the social-cultural context is a construct that encompasses a wide range of factors, including household income, types of income-earning activities, expenditures, physical living conditions, sociodemographic features (including education and family structure), the organization of household management of food acquisition and preparation, and childcare (including patterns of allocation of childcare responsibilities in relation to adult and older children time allocation), as well as many other social characteristics that have been empirically linked to nutrition-related outcomes. Culture refers specifically to the ideational domain of determinants that includes beliefs and values, as well as perceptions and attitudes that affect and relate to the acquisition, preparation, and consumption of food.

The factors that are included in the social-cultural context affect every step in the PIP of a nutritional intervention up to the point at which the nutrients or foods are swallowed by the intended beneficiary. These actions are performed by those who move the elements of the intervention (i.e., information and supplements) through the program to the household and within the household to the child. A PIP diagram alerts us to the fact that all of the actors, in both the program delivery component and the household delivery component, can be regarded as implementors because they have responsibilities to move the elements of the intervention to the intended beneficiaries. All implementors’ behaviors are affected by their social conditions and characteristics and by their cultures—their motivations and beliefs, including the knowledge they acquire from participating in the program. The latter applies not only to the household implementors but also to the delivery implementors. It applies to both simultaneously at the PIP transmission step from program to household (32).

In the case of a nutrition intervention that involves a biological agent, the social-cultural context determines a child's potential to benefit as much as it is determined by the biological efficacy of the agent. This is the case not only for ensuring that the supplement reaches the child's mouth but also for ingesting it (7) no matter how efficacious a supplement is, assessed from a biological perspective that the child cannot benefit if it is not swallowed. The effectiveness of a nutrition intervention that involves a biological agent (e.g., a supplement) thus depends on the effectiveness of all of the elements in both the program delivery system and the household utilization system to ensure that the agent is swallowed. In a nutrition intervention that does not involve an agent but depends on improvements in dietary intake, the pathway to effectiveness is essentially the same. Both the program delivery system and the household delivery system play major roles in ensuring the intervention is consumed. Thus, we can say that a child's “potential to benefit” is the product of the multiplication of the social-cultural potential to benefit times the biological potential to benefit. These, in turn, depend on the biological and social-cultural contexts.

One of the major advantages of an interactive program-research structure, such as the one between Prospera and the INSP research group, is that it permits the sustained linking of a research organization with a program delivery institution and encourages and facilitates problem-solving research to understand and correct bottlenecks and other barriers to program effectiveness. Throughout its history, the research group has been thoughtful and prescient in studying the role of the social-cultural context for the program, which is a major determinant of bottlenecks in the flow of the supplements and the flow of knowledge. As noted above, the latter component was intended to support health and nutrition of the beneficiaries, above and beyond the supplement.

The examination of social-cultural factors in programs is challenging for multiple reasons. One challenge is differentiating between factors that are basically homogeneous across an entire nation and cultural-social factors that differ significantly among subgroups, specifically with respect to their implications for program modifications. Identifying and understanding diversity that matters for programs goes beyond the obvious issue of mutually unintelligible languages. Differences in language are well understood to be a factor that requires attention. However, the challenges for program design go beyond the task of finding linguistic equivalences to use in communicating with mothers and other household implementors (33). Identifying linguistic equivalence is not, itself, a simple matter of picking a good translation from a bilingual dictionary. The concepts that are important to communicate are embedded in organized belief structures (cf. 34, 35). To design effective behavior change communication messages, these belief structures need to be understood and used.

An example of a nongeographic, nonethnic intragroup diversity cultural factor that affects nutrition interventions everywhere is maternal education. Geographic and ethnic cultural features undoubtedly influence the likelihood of a girl receiving more education before she becomes a mother. However, we are also referring here to the broader effects of education, in and of itself, on various nutrition parameters. Throughout the world (e.g., 36) and in Mexico (37), there is strong evidence that this cultural factor has a powerful differential effect on nutrition. This is demonstrated, for example, in the epidemiological research on the impact of education as wealth increases in improving growth in children and preventing obesity in their mothers concurrently (37). At present, there is little in the way of guidance for program development that addresses this issue.

#### Social-cultural context in the Prospera studies

The supplement articles that are particularly important with respect to the INSP research on the social-cultural context (13, 14, 38) provide numerous examples of the value of examining the influences of social-cultural contextual factors.

Beginning with the often-problematic communication component of the “handover” of knowledge between implementors on the delivery side and implementors on the household side (mothers and other child caretakers), the research revealed an underlying problem that originates from social and cultural differences between health workers and caregivers. A primary source of poor communication was that the health workers were trained and oriented to working with patients from the perspective of curing or healing their problems. They were much less experienced or comfortable in providing information and support for “nonmedical actions” that were designed to improve nutrition. Moreover, as a consequence of the emphasis on curative care, training of health workers often did not include adequate knowledge about the technical and epidemiologically documented links between good nutrition, disease prevention, and the role of nutritional status in the efficacy of curative care. Also, in addition to the fact that health workers operate in a curative care environment that implicitly rewards attention to disease management, other social and cultural factors affect the communication between delivery implementors and household implementors. These include social class, education, and belief system differences.

The INSP researchers who focused on social-cultural context factors were highly sensitive to the issues of cultural homogeneity and intracultural/intrapopulation diversity. In the series of studies, Bonvecchio et al. (38) identified and delineated areas of cultural homogeneity (shared cultural elements), as well as features of diversity. For example, with respect to belief system homogeneity, it is intriguing to learn that ideas that entered Mexican culture with the Conquest (e.g., the fundamental components of the “hot-cold system” and the pan–Latin American concept of “empacho”) are found throughout the country and cross-cut ethnic and social class divisions.

The studies reviewed in Bonvecchio et al. (38) also revealed important “heterogeneity” of cultural values across groups of beneficiaries, including features that required program adaptations in recommendations and messages. These essential discoveries concerning subcultural differences were made possible by a research strategy that evolved in relation to the investigators’ attention to factors that led to inadequate flow of the intervention both to households and within them. The research showed that characteristics that matter were not limited to obvious differences in language or geographic features that constrain communication and that also relate to major differences in diet. A dramatic illustration of an ethnic factor that emerged early in the INSP examination of social-culture diversity and adherence to program recommendations for Nutrisano use was the discovery that indigenous women felt it was morally wrong not to share the supplement with other children in the household, in addition to the targeted 6- to 24-mo-old child, not least because it involved overt discrimination between siblings. The message (and the distribution plan) that limited the supplement to children aged 6–24 mo was, therefore, unacceptable in indigenous areas (39).

The framework presented by Bonvecchio et al. (38) lays out a comprehensive guide to 4 major dimensions of the social-cultural context that guided the valuable research of the INSP team over the years. We can postulate that a contributing factor to the positive outcomes of the Prospera program reflects its attention to synergistic social-cultural factors. Among these factors, Mexico's commitment to expanding girls’ access to education has undoubtedly played an important role.

Concluding this section, we note that at present, there are no empirically based guidelines that programs can use to determine, a priori, which factors require careful assessment in any given society. Until such time as these are available, investigators need a body of published work and experience to draw on. Thus, the articles in the supplement constitute an invaluable contribution to the implementation science literature on the roles of the social-cultural context of intervention programs for nutrition.

#### Political context in the Prospera studies

With respect to the political context, the research reported in the supplement bolstered administrative and political commitment to the health component of Prospera. According to Levy (40), the evidence from the evaluations was the primary “political capital” that helped to sustain the program. Thus, it is important to underscore the role the research program played in ensuring program continuity, essentially unchanged, through several changes of governments. This is an unprecedented example of social protection policy continuity in Mexico. However, with the recent government change in Mexico, other political factors have come into play, and the program has undergone substantial modification, with the fate of EsIAN, unfortunately, still not resolved. Paradoxically, across the world, programs that are targeted specifically to improving the health and welfare of the poor are often threatened because of forces that reside in the domain of their political contexts, forces that dictate other priorities.

Another virtue of Prospera has been the transparency of the evaluations. We note, however, with respect to the larger issue of the political context of intervention programs that transparency can also be a threat. We have repeatedly witnessed situations in which imperfections revealed by evaluations are used to condemn a program, even after the imperfections have been addressed, as has happened to Prospera (41). Because of this threat, many program managers resist evaluations.

But, on balance, the social capital gains of follow-up evaluations appear to be usually worth the risk. However, this generalization is only the case when higher-level political goals remain the same as those that led to the design of the original program. In the case of Prospera, the original purpose was mitigating the ill-effects of poverty, including malnutrition. Today, higher-level policy discussions focus on elimination of poverty, not on mitigating its effects. Stepping back to consider the larger picture, it is apparent that constructing a PIP to guide planning and implementation of nutrition interventions should be undertaken with attention to the articulated goals and values that reside in the political context. For example, with respect to EsIAN, a PIP that focuses on its impact on poverty reduction would be the last step of the program implementation pathway.

## Conclusions

To our knowledge, this is the first time that a comprehensive systematic research approach has been developed and applied to support a large-scale nutrition intervention program. Bringing this research together in this supplement is a major contribution to implementation science. It provides knowledge, insight, and inspiration for the future in Mexico and for others elsewhere.

The supplement is a persuasive demonstration of the value of using a PIP model to structure implementation research. It provides an underlying coherence, which is reflected in a supplement that is more than a collection of valuable individual articles.

An important benefit of using a PIP approach in implementation research is that it draws investigators’ attention to the fundamental roles of context and the necessity of identifying, assessing, and addressing context factors and conditions. The articles in the supplement amply demonstrate this, particularly with respect to biological and social-cultural contexts that affected and challenged the effectiveness of Prospera. Identifying the points at which context factors adversely affected or had the potential to adversely affect the program and subjecting the PIP flow data to quantitative and qualitative analyses are models for future implementation research activities. They illustrate the importance of research to anticipate and break bottlenecks in delivery and household utilization systems.

To further use the value of PIP analysis to support the development of more effective interventions, we anticipate that future program-related research in nutrition will include a greater focus on mechanisms than is currently the case. As is widely believed across the range of scientific disciplines, understanding mechanisms permits better design and implementation in the application of science to benefit humankind and, more broadly, the globe in which our species resides. We feel that, at present, mechanisms in intervention research in nutrition programs are generally inadequately examined. For example, as noted above, currently we do not understand the mechanisms through which education of the mother potentiates the effect of wealth on child nutrition. Although this relation is complex, it can be well described mathematically (cf. 36, 37). Similarly, the excellent mathematical prediction of malnutrition's effect on the case-fatality of diseases (42) would be strengthened by persuasive evidence for its mechanisms.

Gaps between empirical description and understanding mechanisms occur in all sciences. Recall that 2 centuries ago, Newton developed excellent predictive equations for the effects of gravity. He was explicit in not attempting to explain the mechanism (*Hypotheses non fingo*) (43), which still eludes physicists despite improvements in mathematical prediction. However, it is essential to recognize that in nutrition, the primary reason for explaining the gaps between epidemiological prediction and understanding mechanisms is the lack of resources to investigate mechanisms. Understanding these mechanisms is essential to improving implementation. Mobilizing resources to begin to close that gap will require advocacy, not only by nutrition professionals and nutrition programs but also in the sectors (e.g., funders and policy makers) on which implementation research depends.

With respect to the biological component of the intervention, the INSP research demonstrates why a probability evaluation to decide whether the distribution of Nutrisano could be stopped was not ethically permissible or feasible. It is highly likely that this type of ethical and feasibility situation will be true for most ongoing nutrition programs throughout the world. Widely implemented vitamin A distribution programs to prevent blindness and child mortality is a salient example of this issue. Since they were initiated decades ago, they have not been scientifically and ethically amenable to examination using conventional evaluation approaches once they were implemented at a national level. However, as Neufeld et al. (26) demonstrate, other scientifically sound methods and approaches can be used to support public health decisions of this nature.

Concerning the INSP research on social and cultural context factors, the journal supplement documents the vital role it played in supporting the health component of Prospera. In addition to its direct programmatic utility, the studies revealed patterns of homogeneity and diversity that are important contributions to the larger body of scientific knowledge on the interactions of social-cultural factors and nutrition. It is a resource that should be widely consulted by research scientists and program planners.

In conclusion, key points we want to highlight are as follows:

1. The considerations and experiences reflected in this journal supplement are important for future program evaluations.
2. The supplement is important as an extraordinarily informative case and is a major contribution to the literature on models of case histories in implementation science in nutrition.
3. The supplement contributes conceptually to implementation research in the clarity of its conceptual organization and in the detailed examination of the many important issues the studies addressed.
4. In our view, every student and practitioner in global and community nutrition should read and ponder the contents of this supplement. We hope it will be recognized as a landmark publication and take its place among the major contributions of scholarly work linking research and action to support programs and improve nutrition in populations.

## Notes

Sources of support: None to declare.

Author disclosures: J-PH and GHP, no conflicts of interest.

Publication costs for this supplement were defrayed in part by the payment of page charges. This article appears as part of the supplement “Implementation Research for Improvement of Nutrition Actions in an At-Scale Social Protection Program in Mexico,” sponsored by the National Institute of Public Health, Mexico (INSP) and the Global Alliance for Improved Nutrition (GAIN). The guest editors of the supplement, Lynnette M Neufeld and Anabelle Bonvecchio Arenas, have the following/no conflicts of interest: LMN is employed by GAIN, and ABA is employed by INSP. The opinions expressed in this publication are those of the authors and are not attributable to the sponsors or the publisher, editor, or editorial board of the *Journal of Nutrition*.

Abbreviations used: C, coverage; E, efficacy; EsIAN, Integrated Strategy for Attention to Nutrition; INSP, National Institute of Public Health of Mexico; PIP, program impact pathway; Q, quality or magnitude of intervention; RCT, randomized controlled trial.
